# Occurrence, Toxicity, Metabolism, Analysis and Control of Mycotoxins—Bridging Knowledge Gaps and Charting Future Directions

**DOI:** 10.3390/toxins18100438

**Published:** 2026-10-11

**Authors:** Ling Wang

**Affiliations:** State Key Laboratory of Rice Biology and Breeding, China National Rice Research Institute, Hangzhou 311401, China; wangling03@caas.cn

## 1. Introduction

Mycotoxins, toxic secondary metabolites produced by filamentous fungi, represent a persistent challenge to global food safety, animal health, and human well-being. With approximately 400 mycotoxins identified to date and an estimated 25% of the world’s food production affected, the economic and public health burden remains substantial. Over the past decades, multidisciplinary efforts have advanced our understanding of mycotoxin biosynthesis, toxicological mechanisms, and mitigation strategies. However, significant knowledge gaps persist regarding fungal–host interactions under changing environmental conditions, the co-occurrence and synergistic effects of multiple mycotoxins, and the translation of laboratory-scale detoxification technologies into practical, scalable interventions.

This Special Issue, “Occurrence, Toxicity, Metabolism, Analysis and Control of Mycotoxins,” was conceived to capture the evolving landscape of mycotoxin research and to address these pressing gaps. A total of 13 papers were published, comprising 11 original research articles and 2 comprehensive reviews, spanning contributions from five continents. Rather than focusing on a single aspect of mycotoxin research, the contributions collected here collectively illustrate how the field is moving towards more predictive, data-driven, and prevention-oriented approaches that ultimately support safer food and feed systems.

## 2. Key Themes and Contributions

### 2.1. Enzymatic and Nanomaterial-Based Detoxification

Enzymatic detoxification has emerged as a promising frontier in mycotoxin mitigation. However, most existing enzymes target single toxins and operate under narrow pH and temperature ranges, limiting their practical application in diverse food and feed processing environments. Addressing these gaps, Gao et al. [1] developed a CotA laccase from *Bacillus subtilis* ZJ-2019-1 capable of simultaneously degrading aflatoxin B1 (AFB1) and zearalenone (ZEN) in both mediator-assisted and immobilization systems, representing a significant step toward multi-toxin remediation using a single enzyme platform. Complementing this work, Dotsenko et al. [2] employed protein engineering to modify zearalenone hydrolase from *Clonostachys rosea*, successfully shifting its optimal pH toward acidic conditions and thereby expanding its applicability in feed processing. At the nanoscale, Mateo et al. [3] provided a comprehensive review of engineered metal nanoparticles, highlighting their dual functionality as antifungal agents and mycotoxin adsorbents. Future research should prioritize the scale-up of these technologies, evaluation of their safety and efficacy in complex food matrices, and pursuit of regulatory pathways for commercial adoption.

### 2.2. Biological Control and Integrated Field Management

Field-level interventions are critical for preventing mycotoxin contamination at the pre-harvest stage. Biocontrol using atoxigenic *Aspergillus flavus* strains has shown promise, yet questions remain regarding its effectiveness under diverse agro-ecological conditions and its impact on non-target mycotoxins. Fundikira et al. [4] evaluated the efficacy of Aflasafe TZ01^®^ in Tanzanian maize and confirmed significant aflatoxin reduction, but also revealed an emerging challenge: increased *Fusarium* mycotoxin contamination following biocontrol application. This finding underscores the need for integrated management strategies that address multiple toxigenic fungi simultaneously. Cebrián et al. [5] took a mechanistic approach by examining the metabolomic response of *Penicillium nordicum* to combined biocontrol agents on dry-cured ham, providing insights into how competitive microbiota suppress ochratoxin A production at the metabolic level. Moving forward, long-term field trials and multi-pathogen surveillance systems will be essential to optimize biocontrol strategies for different cropping systems and climatic zones.

### 2.3. Feed Additives and Livestock Health

Chronic low-level exposure to mycotoxins through contaminated feed poses a significant threat to livestock health and productivity, yet effective countermeasures remain limited. Three contributions to this Special Issue addressed this challenge. Marquis et al. [6] demonstrated that yeast β-glucan supplementation alleviated the negative effects of *Fusarium* mycotoxins on growth performance, intestinal integrity, and liver function in broiler chickens, offering a practical dietary intervention. Priyo et al. [7] investigated difructose anhydride III (DFA III) supplementation in Japanese Black cattle chronically exposed to low levels of zearalenone, showing improvements in serum biochemical parameters. These studies highlight the importance of developing feed additives tailored to real-world, low-level exposure scenarios. Future research should explore the synergistic effects of combined feed additives, assess their long-term safety, and validate their efficacy across different livestock species and production systems.

### 2.4. Human Health Risk Assessment and Emerging Mycotoxins

Understanding mycotoxin exposure in vulnerable populations remains a critical priority, particularly for reproductive-age women, infants, and individuals in low-resource settings. Traditional exposure assessment relying on dietary surveys often underestimates actual intake, highlighting the need for biomarker-based approaches. Szentirmay et al. [8] detected multiple mycotoxins in human follicular fluid and correlated their presence with impaired reproductive outcomes in women undergoing in vitro fertilization, representing a pioneering step in linking mycotoxin exposure to female fertility. Tuba et al. [9] assessed aflatoxin M1 levels in breast milk and urine of nursing mothers in Bangladesh, revealing concerning exposure levels in exclusively breastfed infants and emphasizing the transgenerational dimension of mycotoxin risk.

Beyond classical mycotoxins, emerging toxins are attracting increasing attention. Wang et al. [10] characterized the acute toxicity and hepatotoxicity of ustilaginoidin D, a mycotoxin produced by *Ustilaginoidea virens* (the causal agent of rice false smut), expanding the spectrum of mycotoxins requiring regulatory attention. Rašić et al. [11] explored the cytotoxic and genotoxic interactions between citrinin and cannabidiol in multiple cell lines, addressing the timely issue of mycotoxin contamination in cannabis and hemp products—a rapidly growing market with limited regulatory oversight. These studies call for expanded biomonitoring programs and regulatory frameworks that encompass both established and emerging mycotoxins, particularly in high-risk populations.

### 2.5. Analytical Innovations and Sampling Strategies

Accurate detection and quantification remain foundational to mycotoxin research, yet conventional bulk analysis methods fail to capture the spatial heterogeneity of contamination within a single batch. Teixido-Orries et al. [12] analyzed 14 mycotoxins in 200 individual oat kernels from two contaminated batches, revealing extreme variability in mycotoxin distribution at the single-kernel level. These findings have profound implications for sampling protocols and highlight the limitations of relying on composite samples for risk assessment. Kibugu et al. [13] provided a comprehensive overview of dietary mycotoxins, covering toxicokinetics, toxicodynamics, epidemiology, detection methods, and mitigation strategies, serving as a valuable reference for researchers and policymakers alike. Future analytical developments should prioritize field-deployable sensors, real-time monitoring systems, and harmonized protocols for multi-mycotoxin analysis across different commodity types.

## 3. Knowledge Gaps and Future Directions

Despite the significant advances presented in this Special Issue, several critical knowledge gaps remain.

First, **multi-mycotoxin co-occurrence and synergy** require greater attention. Most studies still focus on individual toxins, yet real-world contamination involves complex mixtures with potentially additive or synergistic effects. The interactive effects of mycotoxins with emerging contaminants, such as cannabinoids [11], represent an underexplored area with growing relevance. Systematic toxicological studies using mixture designs and physiologically based pharmacokinetic modeling are urgently needed.

Second, **sampling and detection methodologies** need refinement. The extreme heterogeneity of mycotoxin distribution demonstrated at the single-kernel level [12] calls for the development of rapid, non-destructive, field-deployable sensing technologies. Artificial intelligence and machine learning approaches, particularly deep learning architectures integrated with hyperspectral imaging, hold transformative potential for real-time quality control along the food supply chain.

Third, **translation from laboratory to field** remains a major hurdle. While enzymatic detoxification shows great promise [1,2], issues of enzyme stability, production cost, and regulatory approval must be addressed before widespread adoption. Similarly, biocontrol products [4] require long-term monitoring to ensure sustained efficacy and ecological safety across diverse agricultural systems.

Fourth, **emerging mycotoxins and exposure routes** warrant systematic surveillance. The identification of ustilaginoidin D as a hepatotoxin [10] and the detection of mycotoxins in follicular fluid [8] suggest that both the spectrum of relevant mycotoxins and the routes of human exposure may be broader than currently recognized. International collaborative networks for emerging mycotoxin surveillance should be established.

Looking ahead, addressing the global challenge of mycotoxins will require continued collaboration among researchers, industry, regulatory authorities, and policymakers. Adopting a **One Health perspective**—recognizing the interconnectedness of agricultural production, environmental change, food safety, and human and animal health—provides an appropriate framework for tackling these complex challenges. Future research should prioritize (i) the development of multi-toxin degradation platforms with industrial scalability; (ii) the integration of omics technologies to elucidate the mechanisms of action of biocontrol agents and feed additives; (iii) large-scale biomonitoring studies in underrepresented regions, particularly in Africa and South Asia; (iv) the application of digital technologies, including artificial intelligence and blockchain-based traceability, for precision mycotoxin management; and (v) climate-responsive risk assessment models that anticipate shifts in mycotoxin occurrence patterns under global warming scenarios.

## 4. Conclusions

This Special Issue has brought together a diverse collection of studies that advance our understanding of mycotoxin occurrence, toxicity, metabolism, analysis, and control. From enzymatic breakthroughs to clinical insights, from field interventions to analytical innovations, the contributions reflect the vitality and breadth of contemporary mycotoxin research. As Guest Editor, I express my sincere gratitude to all authors, reviewers, and editorial staff whose dedication made this collection possible. It is my hope that this Special Issue will not only serve as a snapshot of current progress but also encourage future research that is increasingly predictive, collaborative, and internationally connected. By integrating analytical innovation with toxicological understanding and risk assessment, the mycotoxin research community will be better equipped to protect both human and animal health in an increasingly dynamic global food system.
